# CLARIX FLO Inhibits DRG Adhesion-Induced Neuropathic Pain Through the CD44–TRPV1 Signaling Pathway

**DOI:** 10.3390/ijms27073096

**Published:** 2026-03-28

**Authors:** Chia-Chi Kung, Shih-Ping Dai, Chao-Chiang Tu, Tsung-An Tsai, Po-Heng Chen, Chao-Hsien Sung, Chun-Hsien Fu, Jen-Hao Liu, Chih-Li Chen

**Affiliations:** 1 School of Medicine, College of Medicine, Fu Jen Catholic University, No. 510 Zhongzheng Rd., Xinzhuang Dist., New Taipei 24205, Taiwan; drpainkung@gmail.com (C.-C.K.); 141596@mail.fju.edu.tw (C.-C.T.); aboutdawn0719@gmail.com (P.-H.C.); a02664@mail.fjuh.fju.edu.tw (C.-H.S.); 2 Department of Anesthesiology, Fu Jen Catholic University Hospital, Fu Jen Catholic University, New Taipei 24352, Taiwan; ooo7828@gmail.com (S.-P.D.); na0822@hotmail.com (T.-A.T.); jimmy6291991@gmail.com (C.-H.F.); a04717@mail.fjuh.fju.edu.tw (J.-H.L.); 3 Department of Surgery, Fu Jen Catholic University Hospital, Fu Jen Catholic University, New Taipei 24352, Taiwan

**Keywords:** neuropathic pain, dorsal root ganglion, oxidative regenerative cellulose, anti-inflammation, CLARIX FLO

## Abstract

DRG adhesion is a key pathological feature of failed back surgery syndrome and a major cause of neuropathic pain. DRG, or epidural adhesion, commonly results from spinal surgery, leakage of disk material into the epidural space, or inflammation. To better mimic this clinical condition, we developed a novel and reliable animal model of DRG adhesion-induced neuropathic pain. Using this model, we investigated the therapeutic potential and underlying mechanisms of CLARIX FLO, a sterile, particulate human amniotic membrane and umbilical cord tissue product. Our results demonstrate that CLARIX FLO exerts significant analgesic and anti-inflammatory effects in the DRG adhesion model. The application of CLARIX FLO to the injured DRG markedly attenuated mechanical allodynia. CLARIX FLO treatment also reduced outer sheath thickening, suppressed the inflammatory microenvironment, and decreased hypersensitivity of isolectin B4-positive neurons. Mechanistically, CD44 was identified as a potential downstream mediator of CLARIX FLO. Furthermore, a high dose of HC-HA/PTX3, the key bioactive component of CLARIX FLO, effectively reversed mechanical allodynia and inflammation. Notably, CLARIX FLO inhibited the overexpression of TNF-α and TRPV1 adhering to the DRG. In this study, we demonstrated that CLARIX FLO effectively alleviates DRG adhesion-induced neuropathic pain through a CD44–TRPV1-dependent mechanism.

## 1. Introduction

Neuropathic pain is a complex disorder involving multiple components of the nervous system. Primary afferent nociceptors play a critical role in transmitting sensory information to projection neurons in the dorsal horn [1]. Along this pathway, the peripheral nervous system detects and conveys nociceptive signals, whereas the central nervous system integrates, processes, and modulates both the sensory and affective dimensions of pain [2]. Increasing evidence indicates that neuroinflammation is a key contributor to neuropathic pain. Proteomic profiling by Majumdar et al. demonstrated the upregulation of S100 proteins—particularly S100A8, a mediator of chronic inflammation, and S100B, a marker of brain injury—in the sciatic nerve (SN), spinal cord (SC), and orbitofrontal cortex (OFC). This upregulation coincided with increased expression of GFAP and Iba-1, suggesting astrocyte and microglial activation [3]. Furthermore, mitochondrial dysfunction, impaired autophagy, glial priming, and ion channel dysregulation have been implicated in the pathogenesis of neuropathic pain [4]. Consistently, Chen et al. [5] reported that chronic constriction injury (CCI) induces microglial polarization toward a pro-inflammatory phenotype, accompanied by mitochondrial ROS accumulation and activation of the NLRP3 inflammasome.

Failed back surgery syndrome (FBSS)-associated low back pain, frequently linked to postoperative nerve root adhesion, represents one of the most common complications following spinal surgery. In patients with FBSS, inflammatory changes are predominantly localized to the adhered-to dorsal nerve roots. This observation provides a valuable framework for investigating neuropathic pain induced by dorsal root ganglion (DRG) adhesion. FBSS affects approximately 15–39% of patients and imposes a significant social and economic burden. Previous studies indicate that up to 79% of patients continue to experience chronic or recurrent low back pain for at least 12 months postoperatively [6,7]. Moreover, individuals with persistent low back pain often report substantial pain and disability, with limited functional improvement over time [8].

Adhesion resulting from persistent inflammation around the nerve roots or dorsal root ganglion (DRG) is a major cause of chronic neuropathic pain. Following peripheral injury, continuous noxious stimulation activates sensory neurons and promotes immune cell recruitment, including macrophages and T cells [9,10,11]. These immune cells release pro-inflammatory mediators such as TNF-α, IL-1β, and CCL2, which sensitize nociceptors by upregulating ion channels, including TRPV1 and Nav1.8, thereby enhancing neuronal excitability. Activated nociceptors, in turn, release neuropeptides such as substance P and CGRP that further stimulate immune cells, creating a self-perpetuating neuroimmune feedback loop.

Persistent inflammation and fibrosis around the DRG not only sustain molecular sensitization but also lead to physical adhesion and compression of neural tissue, exacerbating pain hypersensitivity. Given the critical role of the DRG in integrating peripheral inflammation and central sensitization, multiple interventions—including corticosteroids, anti-inflammatory drugs, and anti-adhesion materials—have been attempted. However, their efficacy remains limited, emphasizing the need for novel therapeutic approaches targeting the neuroimmune and fibrotic mechanisms underlying adhesion-induced chronic pain [12,13,14].

CLARIX FLO is a sterile, particulate human amniotic membrane and umbilical cord tissue product that is aseptically processed in compliance with current Good Tissue Practice (cGTP) regulations. It is derived from the same donated human placenta following rigorous donor eligibility determination and tissue suitability screening. Previous studies have demonstrated that CLARIX FLO possesses anti-inflammatory and tissue-regenerative properties. Clinically, it has been reported to exert analgesic effects in patients with pain associated with facet joint syndrome, and intra-articular injection of CLARIX FLO has shown promise in alleviating facet joint pain while reducing opioid consumption [15].

Experimental evidence further supports its potential in pain modulation. Local application of CLARIX FLO has been shown to attenuate established postoperative pain hypersensitivity in mice without the adverse side effects commonly associated with opioid use. This analgesic effect appears to be mediated through direct inhibition of nociceptive dorsal root ganglion (DRG) neurons via a CD44-dependent mechanism [16]. We have successfully developed a novel animal model of neuropathic pain by applying oxidized regenerated cellulose (ORC) to the dorsal root ganglion (DRG), thereby inducing perineural fibrosis without causing significant sensory neuron loss or degeneration [17]. In the present study, we aimed to evaluate the therapeutic effects of CLARIX FLO using this newly established model. Specifically, we sought to elucidate the molecular mechanisms underlying the potential analgesic and anti-inflammatory actions of CLARIX FLO.

## 2. Results

### 2.1. CLARIX FLO Treatment Significantly Inhibits L5 DRG Adhesion-Induced Inflammation and Mechanical Allodynia

We successfully developed a novel animal model of neuropathic pain to mimic FBSS, a common clinical disease [17]. This DRG adhesion model successfully induces both chronic pain and inflammation. To explore the molecular mechanisms, we used CLARIX FLO, a human birth tissue product, as a non-opioid therapeutic, applying it directly to the DRG adhesion site.

We treated the L5 DRG simultaneously with 1 mg of CLARIX FLO and with the application of oxidized regenerated cellulose (ORC). Neither DRG adhesion surgery nor treatment affected mouse body weight (growth of 0.4 to 0.6 g per week) or survival rate (~100%) (Figure S1A,B).

**Figure 1:**
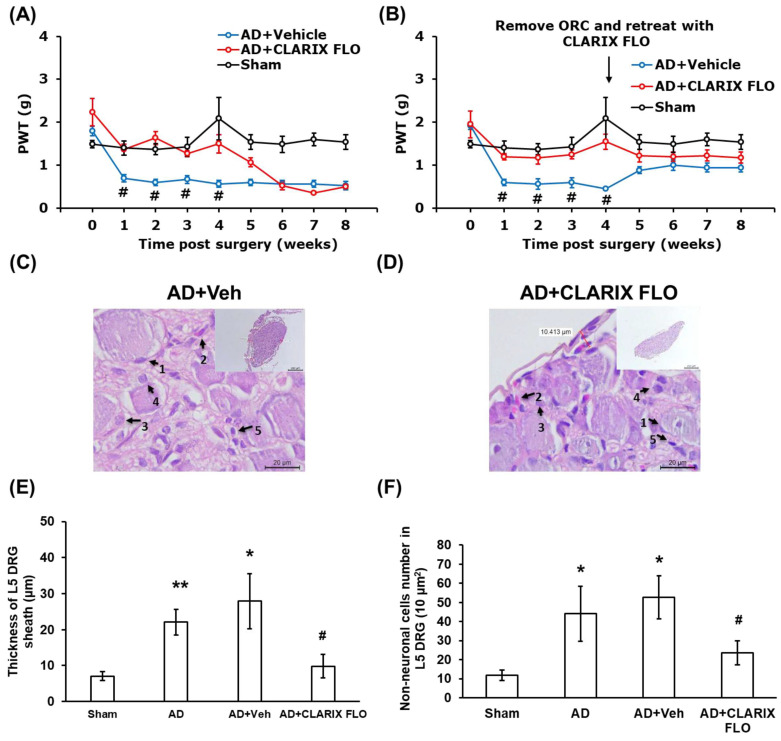
CLARIX FLO reduces DRG adhesion-induced mechanical allodynia, neuron sheath thickening, and non-neuronal cell number. (**A**) Mechanical behavior was measured for 8 weeks after surgery. Comparison between the AD + CLARIX FLO and AD + Vehicle group was achieved using two-way ANOVA with post hoc Bonferroni correction. *N* = 5 for male mice. # *p* < 0.05 (PWT: *p* = 0.043 to 0.044). (**B**) Four weeks after surgery, ORC and adhering connective tissues were removed, followed by the reapplication of 1 mg of CLARIX FLO to lumbar 5 DRG (black arrow point on timeline). A comparison between AD + CLARIX FLO and AD + Vehicle group was performed using two-way ANOVA with post hoc Bonferroni correction (N = 7 to 8 male mice). # *p* < 0.05 (PWT: *p* = 0.032 to 0.046). (**C**,**D**) Representative hematoxylin and eosin (H&E) staining of L5 DRG sections obtained 4 weeks post-surgery. All images were observed using light microscopy. Black arrows indicate non-neuronal cells. Red lines with numbers denote the length of the DRG sheath. Scale bar is 20 μm. (**E**,**F**) Quantification of L5 DRG sheath thickness and non-neuronal cell number in sham (Sham), adhesion (AD), saline-treated (AD + Veh), and CLARIX FLO-treated (AD + CLARIX FLO, 1 mg) groups. (**E**) One-way ANOVA shows a significant difference between groups. Sham vs. other groups, * *p* < 0.05 (*p* = 0.034), ** *p* < 0.01. AD + CLARIX FLO vs. AD + Veh, # *p* < 0.05 (*p* = 0.033). (**F**) One-way ANOVA reveals a significant difference between groups. Sham vs. other groups, * *p* < 0.05 (*p* = 0.041 to 0.043). AD + CLARIX FLO vs. AD + Veh, # *p* < 0.05 (*p* = 0.041). The pictures in C and D are amplification (400×) of parts of the DRG (inserts, 50×). Red line and numbers in the inset of C indicate the thickness of DRG capsule; counterclockwise 32.687; 52.138; 41.567; 28.097; 27.193 9 (μm).

Interestingly, CLARIX FLO treatment significantly reversed the mechanical allodynia induced by the DRG adhesion surgery, with the effect lasting for four weeks (AD + CLARIX FLO: 1.37 ± 0.13 g at 1 W and 1.50 ± 0.2 g at 4 W; AD + Vehicle: 0.70 ± 0.08 g at 1 W and 0.57 ± 0.08 g at 4 W). However, pain recurrence was observed starting at the fifth week post-surgery and sustained until the eighth week (AD + CLARIX FLO: 1.01 ± 0.11 g at 5 W and 0.50 ± 0.04 g at 8 W; AD + Vehicle: 0.60 ± 0.08 g at 5 W and 0.53 ± 0.1 g at 8 W) (Figure 1A).

This suggests that the therapeutic efficacy of CLARIX FLO is sustained for approximately four weeks. To test this hypothesis, we administered a second treatment of 1 mg CLARIX FLO to the L5 DRG at the fourth week after surgically removing the ORC in both the AD + Vehicle and AD + CLARIX FLO groups. The mechanical test showed that this re-treatment extended the analgesic efficacy, which was sustained until the eighth week (AD + CLARIX FLO: 1.2 ± 0.11 g at 5 W and 1.2 ± 0.13 g at 8 W; AD + Vehicle: 0.9 ± 0.07 g at 5 W and 0.94 ± 0.1 g at 8 W) (Figure 1B).

DRG sheath thickening, a characteristic pathological feature of L5 DRG adhesion surgery, is closely associated with neural inflammation. L5 DRG tissues were harvested with or without CLARIX FLO treatment and analyzed using H&E staining (Figure 1C,D). Following CLARIX FLO treatment, the pathological thickening of the DRG sheath was reversed from 22.07 ± 3.52 μm to 9.80 ± 3.26 μm. Furthermore, the vehicle group showed no significant difference compared to the adhesion-only group (AD + Veh: 27.93 ± 7.65 μm) (Figure 1E). We also quantified the non-neuronal cell number (a marker of inflammation). The CLARIX FLO treatment group (AD + CLARIX FLO) exhibited a significant reduction in non-neuronal cells compared to the adhesion-only (AD) and vehicle groups (AD: 44.07 ± 14.45; AD + Veh: 52.60 ± 11.25; AD + CLARIX FLO: 23.61 ± 6.24 per 10 μm2 area) (Figure 1F).

### 2.2. CLARIX FLO Treatment Reduces Inflammation Induced by L5 DRG Adhesion Injury by Modulating Macrophage Polarization

**Figure 2:**
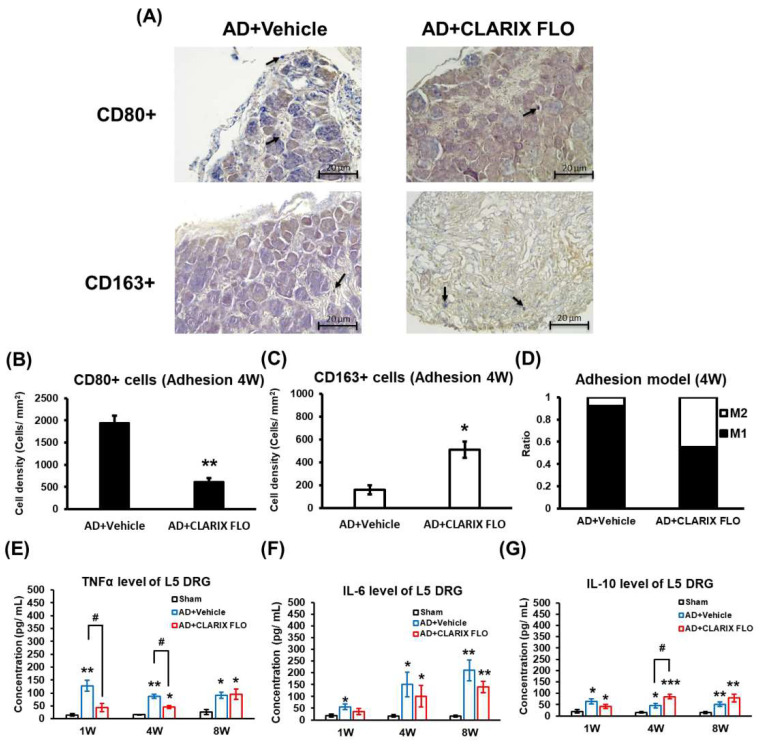
CLARIX FLO reduces inflammation by modulating macrophage polarization in L5 DRG following DRG adhesion surgery. (**A**) Immunohistochemistry staining for CD80 (M1 marker) and CD163 (M2 marker) was performed 4 weeks after surgery. Representative images show macrophage distribution and phenotypes in the L5 DRG. Black arrows indicate CD80- or CD163-positive cells. Scale bar is 20 μm. (**B**–**D**) The histogram illustrates the cell density of macrophages and the M1/M2 ratio in L5 DRG. *n* = 3 male or female mice. Comparison between AD + Vehicle and AD + CLARIX FLO was achieved using an unpaired t-test. * *p* < 0.05, ** *p* < 0.01. (**E**–**G**) L5 DRG tissues were collected 1, 4, and 8 weeks after surgery, respectively. TNFα, IL-6, and IL-10 levels were quantified. Comparisons between experimental groups and the sham were analyzed using the Mann–Whitney U test. * *p* < 0.05, ** *p* < 0.01, *** *p* < 0.001. Comparison between AD + Vehicle and AD + CLARIX FLO was also performed using the Mann–Whitney U test. # *p* < 0.05. N = 3 (repeated three times); n = 5 DRGs per group.

Adhesion of nerve roots induces a local inflammatory condition, leading to the recruitment of macrophages from blood vessels to the injured site. We sought to characterize the macrophage phenotypes within the L5 DRG using CD80 (M1) and CD163 (M2) antibodies. We observed that these macrophages predominantly gathered in the DRG sheath, with only a small population located around the neurons (Figure 2A).

Intriguingly, the CLARIX FLO group exhibited not only a decrease in the number of M1 macrophages but also an increase in the number of M2 macrophages at four weeks (4 W) post-surgery (Figure 2B,C). By combining and normalizing the CD80+ and CD163+ cells in both groups, we demonstrated that CLARIX FLO reverses the inflammatory state by modulating macrophage polarization in the injured L5 DRG (AD + CLARIX FLO, M1:M2 ≈ 55:45; AD + Vehicle, M1:M2 ≈ 92:8) (Figure 2D).

Accumulated evidence indicates that TNF-α contributes to pain pathogenesis, including both neuropathic and inflammatory pain [18,19,20]. Our previous studies showed that this model successfully induces a local increase in TNF-α and IL-6 in the L5 DRG. Here, we verified CLARIX FLO’s anti-inflammatory function by measuring levels of TNF-α, IL-6, and IL-10 in the L5 DRG using ELISA. CLARIX FLO significantly reduced the TNF-α level in the first and fourth weeks post-adhesion but not in the eighth week (Figure 2E). However, CLARIX FLO treatment had no effect on the increase in IL-6 level (Figure 2F). Consistent with M2 polarization, the level of IL-10, an anti-inflammatory cytokine, was significantly increased at the fourth week post-surgery in the CLARIX FLO treatment group (Figure 2G).

### 2.3. CLARIX FLO Treatment Effectively Reversed the Increase in TRPV1and TNFa in the Injured DRG

**Figure 3:**
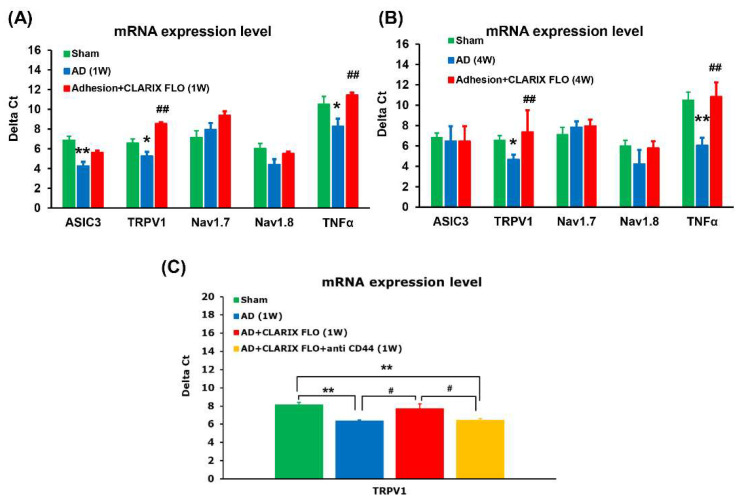
RT-qPCR showed decreased TRPV1 and TNFα expression 1 and 4 weeks post-surgery in the CLARIX FLO group. (**A**,**B**) Ipsilateral L5 DRGs were collected 1 (**A**) or 4 weeks (**B**) post-surgery for RT-qPCR analysis of ASIC3, TRPV1, Nav1.7, Nav1.8, and TNFα mRNA levels. (**A**) Mean ΔCt values 1 week post-surgery were compared via one-way ANOVA with Bonferroni correction. Sham vs. AD: ASIC3 ** *p* = 0.008, TRPV1 * *p* = 0.043, TNFα * *p* = 0.032; Adhesion + CLARIX FLO vs. AD: TRPV1 ## *p* = 0.009, TNFα ## *p* = 0.007. N = 3, n = 6 DRGs/group. (**B**) Mean ΔCt values 4 weeks post-surgery were analyzed via two-way ANOVA with Bonferroni correction. Sham vs. AD: TRPV1 * *p* = 0.044, TNFα *p* = ** 0.007; Adhesion + CLARIX FLO vs. AD: TRPV1 ## *p* = 0.009, TNFα ## *p* = 0.008. N = 3, n = 6 DRGs/group. * *p* < 0.05, ** *p* < 0.01 vs. Sham ## *p* < 0.01 vs. AD. (**C**) Ipsilateral L5 DRGs were collected one-week post-surgery for RT-qPCR analysis of TRPV1 mRNA level. Mean ΔCt values 1 week post-surgery were compared via paired t-test. Sham vs. AD: ** *p* = 0.014; Sham vs. AD + CLARIX FLO + anti CD44: ** *p* = 0.029; AD + CLARIX FLO vs. AD: ^#^ *p* = 0.042; AD + CLARIX FLO vs. AD + CLARIX FLO + anti CD44: # *p* = 0.039. N = 3, n = 6 DRGs/group.

Regarding molecular mechanisms, our findings showed that the expression of TNF-α and TRPV1 was significantly increased in the DRG isolated from the adhesion model compared to the sham control. CLARIX FLO treatment effectively reversed the increase in TRPV1 mRNA after 1 and 4 weeks (1 W, ΔCt, Adhesion: 5.26 ± 0.14 vs. Adhesion + CLARIX FLO: 8.55 ± 0.6; 4 W, ΔCt, Adhesion: 4.68 ± 0.47 vs. Adhesion + CLARIX FLO: 7.37 ± 2.13). TNF-α mRNA was also reduced in the Adhesion + CLARIX FLO group (1 W, ΔCt, Adhesion: 8.27 ± 0.23 vs. Adhesion + CLARIX FLO: 11.45 ± 0.84; 4 W, ΔCt, Adhesion: 6.06 ± 0.75 vs. Adhesion + CLARIX FLO: 10.84 ± 1.41) (Figure 3A,B). Interestingly, CLARIX FLO did not reduce the increase in ASIC3 gene expression, and Nav1.7 and Nav1.8 expression remained unaffected after 1 and 4 weeks. In addition, T-qPCR showed that the CD44 antibody reversed the reduction in the expression of TRPV1 induced by CLARIX FLO treatment adhering to the DRG one-week post-surgery. ΔCt (Sham: 8.27 ± 0.28; AD: 6.36 ± 0.10; AD + CLARIX LFO: 7.71 ± 0.52; AD + CLARIX FLO + anti-CD44: 6.43 ± 0.19) (Figure 3C).

### 2.4. CLARIX FLO Significantly Attenuates Calcium Signal Enhancement in Isolectin B4-Positive Neurons Resulting from L5 DRG Adhesion

The nervous and immune systems are often integrated and coordinated in relation to pain mechanisms [21]. We previously confirmed that the L5 DRG adhesion model enhances acid-induced intracellular Ca2+ signals in injured IB4-positive (IB4+) cells [17]. To connect CLARIX FLO’s anti-inflammatory function with neural activity, L5 DRG tissues treated with CLARIX FLO were harvested 1 and 4 weeks post-surgery and stimulated with an acidic buffer (pH 6.8).

**Figure 4:**
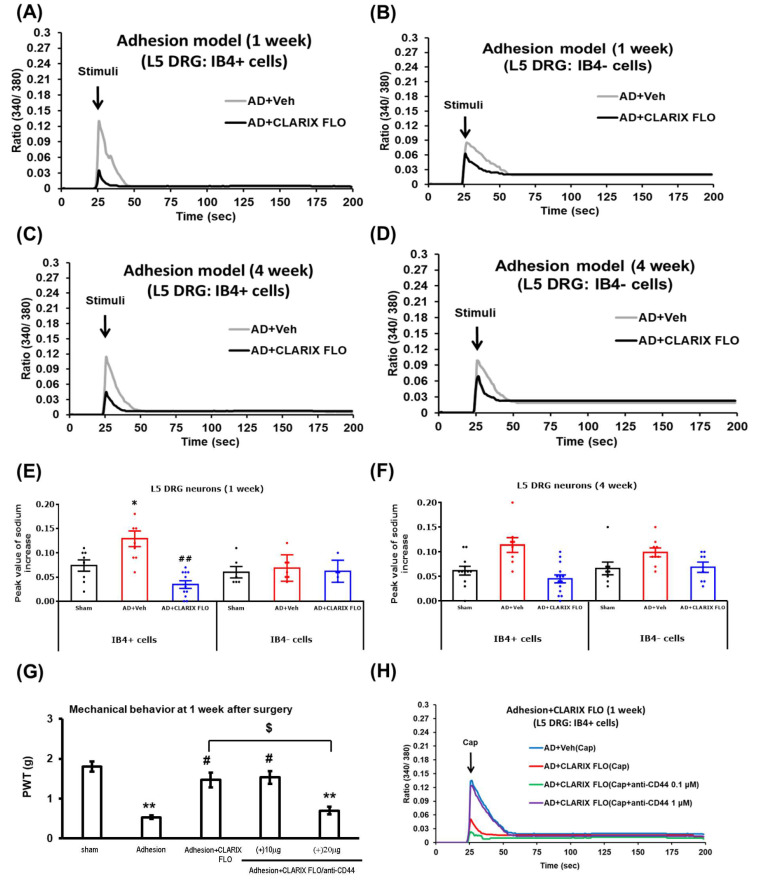
CLARIX FLO attenuates hypersensitization of IB4-positive neurons via CD44 signaling under DRG adhesion conditions. One or four weeks following CLARIX FLO treatment, L5 DRG tissues were collected from mice. (**A**–**D**) The ratio of 340 nm/380 nm fluorescence was used to represent cytosolic calcium levels in IB4-positive (IB4^+^) and IB4-negative (IB4^−^) neurons before and after stimulation with a pH 6.8 acidic buffer. (**E**,**F**) Histograms show the average peak intracellular calcium responses within 15 s after stimulation. Differences in neuronal calcium concentrations between groups were evaluated using the Mann–Whitney U test. * *p* = 0.035, AD + Veh vs. Sham; ## *p* < 0.01, AD + CLARIX FLO vs. AD + Veh. n > 15 total cells per group; mice numbers were 6 to 8. The percentage of responsive neurons in small or medium-sized cells was 75 to 87%. (**G**) L5 DRG was treated with 10 or 20 μg of anti-CD44 antibody (anti-CD44) 25–30 s prior to CLARIX FLO administration [Adhesion (+) CLARIX FLO/anti-CD44]. Mechanical sensitivity was evaluated one week after surgery. Comparison between groups was analyzed via one-way ANOVA. Sham vs. AD + Veh (** *p* < 0.01). Comparison between experimental groups and Adhesion (# *p* = 0.043). Comparison between Adhesion (+) CLARIX FLO/anti-CD44 and Adhesion + CLARIX FLO ($ *p* = 0.041). (**H**) Calcium imaging showed that CLARIX FLO suppresses activation of IB4^+^ neurons via the CD44 pathway. One week after surgery and CLARIX FLO treatment, L5 DRGs were harvested from mice. The 340 nm/380 nm fluorescence ratio indicated cytosolic calcium levels in IB4^+^ cells before and after stimulation with capsaicin (Cap, 0.3 μM). The green and purple traces represent IB4^+^ neurons treated with capsaicin alone and in combination with anti-CD44, respectively.

As anticipated, the increase in calcium levels was reduced in the IB4+ cells but not in the IB4-negative (IB4-) cells (Figure 4A–D). The average highest peak values of the intracellular calcium response (340/380 ratio) in IB4+ cells showed a significant difference between the Adhesion + CLARIX FLO group and the Adhesion + Vehicle group at both Week 1 (AD + CLARIX FLO: 0.03 ± 0.007; AD + Veh: 0.13 ± 0.016) and Week 4 (AD + CLARIX FLO: 0.05 ± 0.008; AD + Veh: 0.11 ± 0.015). Conversely, no difference in calcium level change was observed in the IB4- cells between the two groups (Figure 4E,F).

### 2.5. CLARIX FLO Attenuates the Capsaicin-Mediated Calcium Elevation in Isolectin B4-Positive Cells via CD44-Dependent Signaling

Furthermore, a recent report by Dr. Zhang’s group demonstrated that CLARIX FLO inhibits post-surgical pain via CD44-dependent signaling [16]. We applied 10 or 20 μg of an anti-CD44 antibody along with CLARIX FLO to the L5 DRG prior to ORC application. One week after surgery, treatment with 20 μg of the anti-CD44 antibody significantly reversed the mechanical allodynia reduction provided by CLARIX FLO (Figure 4G). However, 10 μg of the anti-CD44 antibody had no effect on CLARIX FLO’s analgesic function. Additionally, in vitro calcium signaling experiments confirmed that adding 1 μM of the anti-CD44 antibody to L5 DRG IB4+ cells significantly reversed the calcium concentration reduction provided by CLARIX FLO one-week post-adhesion surgery (Figure 4H). Therefore, we conclude that CLARIX FLO reduces L5 adhesion-induced neuropathic pain through a CD44-dependent signaling pathway.

### 2.6. HHP Mitigates L5 DRG Adhesion Surgery-Induced Mechanical Allodynia via CD44-Dependent Signaling

HHP is the key active component of CLARIX FLO. We tested the function of HHP in the L5 DRG adhesion model. Intrathecal injections of 10 or 30 μg of HHP 10 min post-surgery showed no effect on L5 DRG-induced mechanical allodynia.

**Figure 5:**
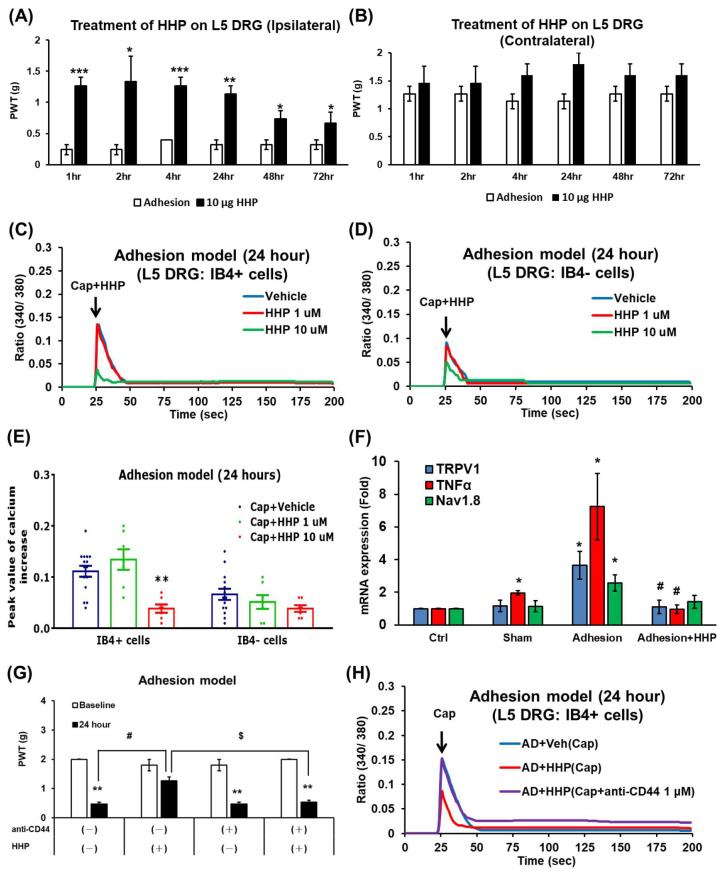
HC-HA/PTX3, the primary component of CLARIX FLO, exerts an analgesic effect by suppressing TRPV1 expression and activity. (**A**,**B**) Mechanical sensitivity was measured 1, 2, 4, 24, 28, and 72 h post-surgery after applying 10 μg of HC-HA/PTX3 to the L5 DRG. n = 3 male or female mice. * *p* < 0.05, ** *p* < 0.01, *** *p* < 0.001, adhesion group vs. 10 μg HHP group via two-way ANOVA. (**C**,**D**) The 340 nm/380 nm fluorescence ratio reflected cytosolic calcium levels in IB4^+^ cells before and after stimulation with capsaicin (Cap, 0.3 μM). (**E**) Histograms show the average peak intracellular calcium responses within 15 s following stimulation. ** *p* < 0.01, Cap + HHP 10μM group vs. Cap + Vehicle group. n > 15 total cells per group; mice numbers were 6 to 8. The percentage of responsive neurons in small or medium-sized cells was 75 to 80%. (**F**) TRPV1, Nav1.8, and TNFα mRNA levels were measured via RT-qPCR in L5 DRGs 24 h post-surgery. N = 3; n = 6 DRGs per group. * *p* < 0.05, adhesion group vs. Ctrl group, # *p* < 0.05, Adhesion + HHP vs. Adhesion group using two-way ANOVA. (**G**) Mechanical sensitivity was measured 24 h post-surgery following application of HC-HA/PTX3 with or without 10 μg of anti-CD44 to the L5 DRG. Comparison among these groups was performed using two-way ANOVA (24-h vs. Baseline ** *p* < 0.01. anti-CD44(−)/HHP (+) vs. anti-CD44(−)/HHP (−) # *p* = 0.039. anti-CD44(+)/HHP (+) vs. anti-CD44(−)/HHP (+) $ *p* = 0.041). (**H**) Calcium imaging showed that HC-HA/PTX3 suppresses activation of IB4^+^ neurons via the CD44 pathway. L5 DRG was collected from mice 24 h after surgery (AD + HHP or AD + Veh) for primary culture. The ratio of 340 nm/380 nm represented the cytosolic calcium level of IB4+ cells before and after capsaicin (Cap, 0.3 μM) stimuli. The purple group/line represents the IB4+ neuron treated with capsaicin and anti-CD44.

However, when 10 μg of HHP was applied directly to the L5 DRG along with ORC, it showed a significant effect in reducing mechanical allodynia for three days. The analgesic effect of the 10 μg HHP group began at 1 h (1.26 ± 0.13 g) and lasted up to 72 h (0.67 ± 0.18 g) (Figure 5A). HHP did not affect the paw withdrawal thresholds (PWTs) of the contralateral paw (Figure 5B). This suggests that the therapeutic efficacy of HHP only lasts for approximately 72 h.

In the in vitro experiments, 1 or 10 μM of HHP was added to cells stimulated with capsaicin 24 h post-surgery. As expected, the increased calcium levels were reduced in the IB4+ cells but not in the IB4- cells (Figure 5C,D). The average highest peak values of the intracellular calcium response (340/380 ratio) in IB4+ cells showed a significant difference between the Cap + HHP 10 μM group and the Cap + Vehicle group at 24 h (Cap + HHP 10 μM: 0.04 ± 0.006; Cap + Vehicle: 0.14 ± 0.009) (Figure 5E). HHP treatment also reversed the increase in TRPV1 and TNF-α mRNA expression at 24 h post-surgery (Figure 5F). Although TNF-α mRNA expression was slightly increased in the sham group, we considered this minor change irrelevant to our hypothesis.

Similarly, to CLARIX FLO, HHP’s mechanism is considered CD44-dependent. Treatment with 10 μg of the anti-CD44 antibody reversed the analgesic effects of HHP on L5 DRG-induced mechanical allodynia 24 h post-surgery (anti-CD44-HHP−: 0.47 ± 0.07 g; anti-CD44-HHP+: 1.27 ± 0.13 g; anti-CD44 + HHP−: 0.47 ± 0.07 g, anti-CD44 + HHP+: 0.53 ± 0.07 g) (Figure 5G). In vitro data further confirmed that adding 1 μM of the anti-CD44 antibody to L5 DRG IB4+ cells significantly reversed the calcium concentration reduction caused by HHP at 24 h post-adhesion surgery (Figure 5H). Additionally, there was no difference in the calcium levels of IB4- cells among the groups 24 h after DRG adhesion surgery (Figure S2B).

### 2.7. HHP Attenuates the Elevation of Pro-Inflammatory Cytokines While Promoting the Induction of Anti-Inflammatory Cytokines

**Figure 6:**
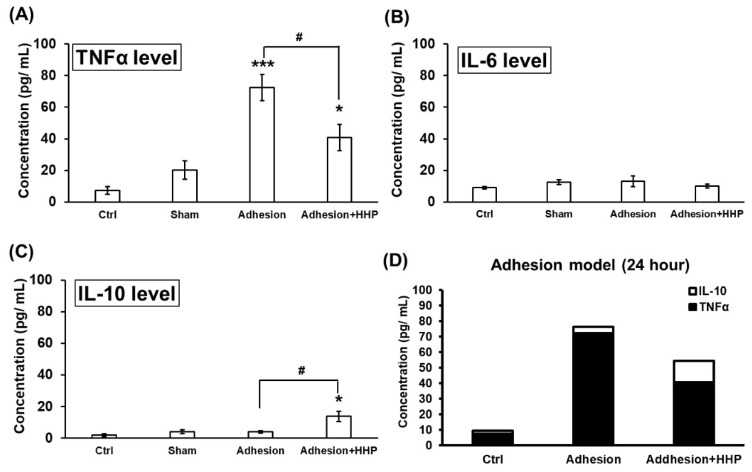
HC-HA/PTX3 reduces inflammation by modulating the TNFα/IL-10 ratio. (**A**–**C**) ELISA was performed to measure TNFα, IL-6, and IL-10 levels in L5 DRGs collected 24 h after surgery. Comparison between experimental groups and Ctrl groups was achieved via a Mann–Whitney *U test*. * *p* < 0.05, *** *p* < 0.001. Comparison between Adhesion and Adhesion + HHP was also carried out via a Mann–Whitney U test. # *p* < 0.05. N = 3, n = 5 DRGs per group. (**D**) The diagram shows the relative proportion of TNFα to IL-10 among different groups.

ELISA analysis confirmed that HHP slightly reduced the TNF-α level at 24 h post-surgery (Adhesion + HHP vs. Adhesion: 40.78 ± 8.33 pg/mL vs. 72.37 ± 8.30 pg/mL) (Figure 6A) and IL-10 level, an anti-inflammatory cytokine produced by M2 macrophages, was slightly increased at 24 h after surgery in the HHP treatment group (Figure 6C), although it had no effect on the IL-6 level (Figure 6B). The resulting histogram demonstrated that HHP treatment could modulate the balance and proportion of cytokines (Figure 6D). Therefore, we propose that HHP plays a role similar to CLARIX FLO in mitigating L5 DRG adhesion-induced neuropathic pain.

## 3. Discussion

In this study, we utilized a well-established animal model that mimics failed back surgery syndrome (FBSS)-induced chronic neuropathic pain to evaluate the therapeutic efficacy of CLARIX FLO. Compared to chronic constriction injury [22], spared nerve injury [23], and spinal nerve ligation [24], the L5 dorsal root ganglion (DRG) adhesion model effectively induces neuropathic pain characterized by directly targeting the DRG but not the sciatic nerve or spinal nerve. Most importantly, the L5 dorsal root ganglion (DRG) adhesion model effectively induces neuropathic pain characterized by persistent inflammation and hyperalgesia, without causing Wallerian degeneration [22]. This finding is more relevant to patients suffering from clinical symptoms of neuropathic pain. Moreover, specific genes identified in this model may serve as potential targets for neuropathic pain treatment. However, the duration and efficacy of current pharmacological interventions remain limited [17], and the detailed mechanisms underlying the L5 DRG adhesion model are still not fully understood.

CLARIX FLO is a sterile, micronized, and lyophilized form of human AM/UC matrix used for surgical and non-surgical repair, reconstruction, or replacement of soft tissue by filling in the connective tissue void. It has been demonstrated to promote regenerative healing through its anti-inflammatory and anti-scarring properties in ophthalmic applications [25,26]. Moreover, CLARIX FLO has also been demonstrated to relieve pain effectively in patients with osteoarthritis [27].

Our study showed that CLARIX FLO prevented the adherence of L5 DRG and mechanical allodynia caused by ORC application (Figure 1A,D). According to Dr. Zhang’s group, CLARIX FLO attenuates heat hypersensitivity in the hind paw when the plantar incision is performed dose-dependently (0.1–0.5 mg). Furthermore, the effectiveness of CLARIX FLO only lasts for four hours [16]. Consistent with their results, a pain relief effect was observed in the CLARIX FLO treatment group for four weeks (Figure 1A). When we re-treated the L5 DRG with CLARIX FLO in the fourth week after surgery, the mechanical test showed that the analgesic efficacy of CLARIX FLO was sustained up to the eighth week (Figure 1B). Since nociceptor hyperexcitability contributes to the transition from acute to chronic pain [28], the cellular and molecular mechanisms underlying CLARIX FLO-mediated pain inhibition may in part be attributed to its modulation of neuronal excitability. Our previous studies suggested that adhesion-induced calcium influx into IB4-positive (IB4^+^) neurons contributes to neuronal hypersensitivity and exacerbates pain development [17]. In the present study, CLARIX FLO treatment significantly attenuated the pH 6.8 acidic buffer-induced increase in intracellular calcium levels in IB4^+^ neurons, which are typically hyperexcitable at both the first and fourth weeks after surgery (Figure 4A–F). In contrast, calcium influx was not elevated in IB4-negative (IB4^−^) neurons (Figure S2A,B). These findings suggest that CLARIX FLO may alleviate L5 DRG adhesion-induced neuropathic pain by suppressing the hyperactivity of IB4^+^ nociceptors. CLARIX FLO also shows anti-inflammation in injured DRG under L5 DRG adhesion injury. DRG sheath thickening, immune cell recruitment, and inflammatory cytokine release are the major characteristics of the L5 DRG adhesion model. In this study, CLARIX not only reduced the DRG sheath thickening (Figure 1E) but also changed the macrophage polarization (Figure 2A–D). The total number of macrophages recruited from blood vessels to the injury site remained consistent across all groups [29]. In the L5 DRG adhesion model, chronic inflammation was induced, resulting in the persistent accumulation of pro-inflammatory M1 macrophages at the injury site. Notably, CLARIX FLO treatment modulated the ratio of M1 to M2 macrophages, as evidenced by an altered M1/M2 ratio (Figure 2D). Consistent with the change in the M1/M2 ratio, CLARIX FLO treatment not only reduced TNFα levels but also increased interleukin-10 (IL-10) expression (Figure 2E,G).

We propose that the observed decrease in TNF-α, together with the increase in IL-10, is largely mediated by the effects of CLARIX FLO and HHP on macrophage polarization, promoting a phenotypic shift from the pro-inflammatory M1 state to the anti-inflammatory M2 state. This transition is critical, as cytokines produced by M2 macrophages are known to suppress TRPV1 sensitization and facilitate tissue repair. In contrast, the dynamics of IL-6 appear more complex. As a pleiotropic cytokine, the pro-inflammatory activity of IL-6 is strongly influenced by the local inflammatory environment. Although IL-6 levels remained relatively stable during the first four weeks of CLARIX FLO treatment—suggesting a temporary homeostatic balance—this protective effect appeared to decline thereafter. We speculate that persistent adhesion surrounding the DRG serves as a continuous noxious stimulus that eventually exceeds the therapeutic capacity of CLARIX FLO. This sustained adhesion may perpetuate chronic inflammation, resulting in late-stage elevation of IL-6 and potential reactivation of nociceptive signaling, similar to the inflammatory progression observed in rheumatoid arthritis [30].

Macrophage polarization occurs alongside a consistent recruitment of macrophages from blood vessels to the injured area, reflecting a stable and coordinated immune response that supports tissue repair. This process is essential for wound healing, as macrophages remove cellular debris and promote angiogenesis and tissue regeneration. The consistency of macrophage recruitment is therefore critical for the proper progression of healing phases. Previous studies have shown that the modulation of the M1/M2 macrophage ratio can influence the transition between the early and late phases of mechanical allodynia in rheumatoid arthritis and chronic constriction injury (CCI)-induced neuropathic pain models [30,31]. Thus, in the present study, CLARIX FLO exerts anti-inflammatory, tissue-protective, and pro-regenerative effects by regulating M1/M2 macrophage polarization.

HHP is the key bioactive component of CLARIX FLO, purified from the water-soluble extract of human amniotic membrane. HHP exhibits anti-inflammatory, anti-scarring, and anti-angiogenic effects [32]. According to Zhang et al. [16], HHP exerts similar functions to CLARIX FLO in nociceptive pain models. In the present study, we also applied HHP to the L5 DRG adhesion model. Our results demonstrated that treatment with 10 μg of HHP at the injury site significantly reduced mechanical allodynia (Figure 5A). However, intrathecal administration of HHP did not lead to any analgesic effect, likely due to the presence of the blood–brain barrier. Because inflammation occurs locally within the L5 DRG, systemic or central delivery of HHP may not effectively target the affected region. Moreover, HHP reduced IB4-positive neuronal activity and downregulated the expression levels of TRPV1 and TNFα, similar to the effects observed with CLARIX FLO treatment (Figure 5C–F). Nevertheless, the duration of the analgesic effect of HHP was notably shorter than that of CLARIX FLO (HHP: 72 h vs. CLARIX FLO: 4 weeks). The difference in the duration of therapeutic effects observed between CLARIX FLO and HHP is likely attributable to the different doses used in our experiments (1 mg for CLARIX FLO vs. 10 μg for HHP).

Cluster of differentiation 44 (CD44) is a multifunctional transmembrane glycoprotein that acts as a cell surface adhesion molecule, regulating a wide range of physiological and pathological processes [33,34]. Hyaluronic acid (HA), a naturally occurring glycosaminoglycan, is well known for its ability to bind to the CD44 receptor, particularly on classically activated M1 macrophages. The injection of TNFα or interleukin-6 into the peripheral system has revealed that both the pronociceptive and antinociceptive effects of HA are mediated through the activation of CD44-dependent signaling pathways in nociceptors [35].

In the present study, treatment with an anti-CD44 antibody at the L5 DRG reversed the analgesic effects induced by both CLARIX FLO and HHP (Figure 4G and Figure 5G). In vitro, capsaicin (TRPV1 agonist)-induced calcium influx was significantly inhibited by CLARIX FLO and HHP; however, this inhibitory effect was abolished when cells were co-treated with the anti-CD44 antibody (Figure 4H and Figure 5H). We also found that CLARIX FLO specifically reduced TRPV1 mRNA expression (Figure 3A,B). These findings suggest that both CLARIX FLO and HHP alleviate L5 DRG adhesion-induced neuropathic pain primarily through the CD44-TRPV1 signaling pathway. Our results also show that the CD44 antibody reversed the reduction in TRPV1 expression induced by CLARIX FLO treatment adhering to the DRG one-week post-surgery (Figure 3C). Consistently, calcium imaging demonstrated that the anti-CD44 antibody significantly reversed the inhibitory effect of HHP on capsaicin-induced calcium influx (Figure 5H). These results suggest that CLARIX FLO/HHP suppresses TRPV1 through a CD44-dependent mechanism. Supporting this interpretation, hyaluronic acid (HA), a CD44 ligand, has been reported to reduce TRPV1-mediated impulse firing and channel sensitization induced by bradykinin [36]. HA also interacts with membrane receptors such as CD44 to regulate multiple cellular processes, including migration, apoptosis, survival, and inflammation [37]. Studies are ongoing to further define the molecular signaling linking CD44 activation to TRPV1 regulation.

It remains unclear whether the CLARIX FLO–CD44-mediated shift in macrophage polarization from the M1 to M2 phenotype is dependent on TRPV1. In our ongoing studies (for next manuscript), DRG adhesion-induced neuropathic pain (allodynia) was attenuated in TRPV1 knockout mice; however, no significant changes in inflammatory status were observed under DRG adhesion conditions in these mice. These findings are consistent with previous reports [38,39,40]. However, CLARIX FLO or HC-HA-PTX3 indeed suppresses TNF-α production through modulation of macrophage polarization. The CD44 signaling pathway has been shown to regulate inflammatory responses by inhibiting NF-κB signaling, a key pathway governing M1/M2 polarization [41]. In the literature, the anti-inflammatory and protective roles of CD44 are largely attributed to its capacity to resolve inflammation via ligand clearance and maintenance of tissue homeostasis [42]. Notably, while CD44 is implicated in the initiation of arthritis, its genetic deletion in mouse models paradoxically exacerbates disease severity rather than alleviating it [43].

Overall, our study demonstrates that CLARIX FLO and its key component, HHP, effectively inhibit inflammation and nociception caused by L5 DRG adhesion. Moreover, we propose a potential mechanistic pathway—HHP–CD44–TRPV1 signaling—as a novel target for treating FBSS-induced low back pain. These findings provide valuable insights for future basic research and clinical applications.

## 4. Materials and Methods

### 4.1. Animals

Wild-type male or female C57BL/6 or ICR mice (8 weeks old, 20–25 g) were obtained from BioLASCO Taiwan (Taipei) and individually housed in cages under controlled temperature and humidity conditions with a 12 h light/dark cycle. Food and water were provided ad libitum at the Laboratory Animal Center of Fu Jen Catholic University. All animal experiments were carried out in accordance with the Guide for the Care and Use of Laboratory Animals (U.S. National Research Council) and approved by the Institutional Animal Care and Use Committee (IACUC) of Fu Jen Catholic University, Taiwan. Care was taken to ensure the well-being of the mice throughout the study. The animals’ behavior was monitored for eight weeks following the establishment of the models. Inflammatory cytokines and pathology were evaluated on the 1st, 4th, or 8th week after adhesion. Neuronal activity was assessed by measuring calcium signals in L5 DRG neurons 1 and 4 weeks after adhesion.

### 4.2. Experimental Agents

For the in vivo experiments, all drugs or peptides were diluted in saline prior to injection. For the in vitro trials, all drugs or peptides were diluted in HEPES/MES buffer (125 mM NaCl, 1 mM CaCl2, 1 mM MgCl2, 8 mM glucose, 10 mM HEPES, and 15 mM MES, pH 7.6). Chemicals: CLARIX FLO and HHP were purchased from BioTissue (Miami, FL, USA). SURGICEL^®^ Absorbable Hemostat (oxidized regenerated cellulose, 5 cm × 35 cm) was bought from ETHICON (Somerville, NJ, USA). Rabbit polyclonal anti-CD80 antibody (Cat.No. ORB5805) and rabbit polyclonal anti-CD163 antibody (Cat.No. ORB13303) were purchased from Biorbyt (Cambridge, UK). Fura-2-AM Dye (Cat.No. 65-0858-39) was obtained from Thermo Fisher (Waltham, MA, USA). Capsaicin (Cat.No. M2028) was purchased from Sigma-Aldrich (St. Louis, MO, USA). CD44 monoclonal antibody (Cat.No. 60224-1-lg) was acquired from Proteintech (Rosemont, IL, USA).

Enzyme-linked immunosorbent assay (ELISA) kits for tumor necrosis factor α (TNF-α) (Cat.No. MTA00B), interleukin 6 (IL-6) (Cat. No. M6000B), and interleukin 10 (IL-10) (Cat.No. M1000B) were purchased from R&D Systems (Minneapolis, MN, USA).

### 4.3. Surgical Procedure and Drug Treatment

All mice were anesthetized with 1.5% isoflurane (Forane^®^; AbbVie Ltd., Berkshire, UK). The skin over the back and right-side latissimus dorsi (ipsilateral site) was slit. After cleaning the fleshes around the lumbar vertebra, the fifth lumbar dorsal root ganglion (L5 DRG) was exposed using microsurgical instruments. The exposed L5 DRG of mice was then covered with 0.8–1.0 mg of SURGICEL^®^ Absorbable Hemostat (oxidized regenerated cellulose, ORC) (Johnson & Johnson MedTech, NJ, USA). After surgery, the muscle and skin were sutured using a surgical suture. In the sham-operated group, the L5 DRG was exposed to, but not covered with, ORC. In the drug-treated group, 1 mg of CLARIX FLO or 10 μg of HHP mixed in 10 μL of 0.9% saline was applied to the exposed L5 DRG of mice prior to covering with ORC.

### 4.4. Behavioral Test

Behavioral testing was performed between 9:00 am and 5:00 pm. An effort was made to minimize the number of animals used and their suffering. For mechanical sensitivity testing, male C57BL/6 mice were evaluated for paw withdrawal thresholds to calibrate von Frey filaments (Touch-Test, North Coast Medical, Morgan Hill, CA, USA) applied to the plantar surface of the hind paw. Mice were habituated to the testing environment for 1–2 h per day for two consecutive days before data collection. During testing, each filament was applied five times at 5 s intervals to each hind paw. PWT was defined as the minimum force that elicited paw withdrawal in at least three out of five stimulations. For thermal testing, mice were allowed to acclimatize to the testing apparatus at least 2 h before assessment, while in experiments, the plantar surface of each hind paw was exposed to radiant heat (30% light intensity). The latency to withdrawal of the paw from radiant heat was recorded. Three measurements were taken at 1 min intervals for each paw, and the mean value was used for analysis.

### 4.5. Hematoxylin and Eosin and Immunohistochemistry Staining

For histological analysis, the L5 dorsal root ganglion (DRG) was collected from each mouse 4 weeks after surgery. Tissues were fixed, embedded in paraffin, sectioned, and stained with hematoxylin and eosin (H&E) for morphological evaluation (H&E, served by Array Biotechnology Co., Ltd. Republic of China (Taiwan)). Stained sections were observed using the Leica DM500 Microscope (40×) (Wetzlar, German) and analyzed using LAS V4.12 software (Thermo Fisher Scientific, MA, USA). The thickness of the DRG outer sheath and the number of non-neuronal cells were quantified. The scale bar represents 20 μm. For immunohistochemical staining, paraffin-embedded sections of the L5 DRG were deparaffinized and rehydrated through graded ethanol. Sections were then blocked for 2 h at room temperature. After blocking, primary antibodies against CD80 (2 μg/mL) or CD163 (5 μg/mL) were applied, and the slides were incubated at 4 °C overnight (16 h). Following incubation, slides were washed and then incubated with the appropriate secondary antibody for 1 h at room temperature. After washing, color development was performed using BCIP/NBT substrate. Finally, slides were mounted with mounting medium and covered with a coverslip for microscopic observation.

### 4.6. TNFα, Interleukin 6, and Interleukin 10 Level Measurement

The L5 dorsal root ganglia (DRG) were harvested from mice on the 1st, 4th, or 8th week after surgery, homogenized in tissue lysis buffer (RIPA buffer), and centrifuged at 10,000× *g* for 10 min at 4 °C (Allegra 64R Centrifuge, Beckman Coulter, CA, USA). The levels of TNF, IL-6, and IL-10 were determined using ELISA kits (R&D Systems).

### 4.7. Primary Culture and Calcium Imaging

The ipsilateral L5 dorsal root ganglia (DRG) were isolated from mice sacrificed after 24 h or the 1st or 4th week after surgery. The DRGs were incubated in a collagenase–DMEM mixture (50 μL of 20× collagenase diluted in 950 μL of serum-free DMEM) at 37 °C for at least 1 h, with gentle mixing every 15 min (five times in total). After incubation, 1 mL of 0.25% trypsin was added, and the tissue was dissociated via gentle pipetting. The samples were incubated again under the same conditions and gently agitated every 5 min for 15 min. Following digestion, 6 mL of primary culture medium was added, and the samples were centrifuged at 1000 rpm for 5 min. The supernatant was discarded, and the cell pellet was resuspended in 50 μL of primary culture medium. The final cell suspension was equally distributed onto poly-L-lysine-coated coverslips (each ipsilateral or contralateral sample was divided into two coverslips, 50 μL per coverslip). After incubating the primary culture cells at 37 °C for 1.5–2 h, 2 mL of primary culture medium was added to each 3.5 cm dish. After 12 h of incubation, the primary-cultured neurons and their medium were gently replaced with 1 mL of serum-free DMEM containing 1.3 μM Fura-2 AM (Molecular Probes, Thermo Fisher Scientific, MA, USA). The cells were then incubated at 37 °C for 30 min in the dark. Following incubation, the medium was replaced with 300 μL of pH 7.6 balancing buffer in the imaging chamber (125 mM NaCl, 1 mM KCl, 1 mM MgCl_2_, 5 mM CaCl_2_, 8 mM glucose, 10 mM HEPES, and 15 mM MES) for observation under an inverted fluorescence microscope (Leica DMI3000B, Wetzlar, German). A 63× objective lens was used for high-magnification imaging. The fluorescein isothiocyanate (FITC) channel was applied to detect IB4-FITC fluorescence. Changes in intracellular calcium concentration were monitored using a high-speed wavelength switcher (Lambda DG4, Thermo Fisher Scientific, MA, USA), which rapidly alternated the excitation wavelengths between 340 nm and 380 nm. The emitted fluorescence intensity was recorded, and the ratio of 340/380 nm was analyzed using Metafluor software (version 7.5, Thermo Fisher Scientific, MA, USA). During the calcium imaging experiment, neuronal calcium activity was recorded for a total duration of 200 s. At the 25th second, DRG neurons were stimulated with either acidic buffer (600 μL, adjusted to pH 6.8) or 0.3 μM capsaicin. The highest calcium response peak observed between 25 and 40 s was selected for analysis. Following the recording of calcium responses to buffer stimulation, the medium was replaced with pH 7.6 balancing buffer containing 0.7 mg/mL IB4-FITC (Sigma, MO, USA) and the cells were incubated for 5 min. IB4-positive (IB4^+^) neurons were then identified and quantified using MetaView software (version 7.5, Thermo Fisher Scientific, MA, USA).

### 4.8. Quantitative RT-PCR

L5 DRGs were harvested and pooled in either the 1st or 4th week after surgery for RNA extraction. Each RNA sample was prepared from a pool of three DRGs collected from three mice. Total RNA was extracted using the RNeasy Kit (Qiagen, Valencia, CA, USA). For quantitative PCR, gene-specific primers (100 nM), cDNA templates, and master mix containing SYBR Green I and AmpliTaq Gold DNA Polymerase (Applied Biosystems, Foster City, CA, USA) were combined, and reactions were performed using an ABI Prism 7300 system. Each assay was conducted in triplicate. Thermal cycling conditions consisted of an initial denaturation at 95 °C for 10 min, followed by 40 cycles of 95 °C for 15 s and 60 °C for 1 min. The threshold cycle (Ct) values for both the target genes and the internal control (mGAPDH) were obtained from the same samples, and relative gene expression was calculated using the comparative Ct (ΔΔCt) method. The primer sequences were as follows: TRPV1: 5′-tctccactggtgttgagacg-3′ (forward)/5′-gggtctttgaactcgctgtc-3′ (reverse) ASIC3: 5′-tttcacctgtcttggctcct-3′ (forward)/5′-caggatagtggtggggattg-3′ (reverse) Nav1.7: 5′-caacgcactcataggagcaa-3′ (forward)/5′-cttgccagcaaacagattgac-3′ (reverse)Nav1.8: 5′-gtagtggtggatgccttggt-3′ (forward)/5′-aagtggccggtattgttttg-3′ (reverse)TNFα: 5′-ggtgcctatgtctcagcctctttt-3′ (forward)/5′-gccatagaactgatgagagggag-3′ (reverse)GAPDH: 5′-ggagccaaacgggtcatcatctc-3′ (forward)/5′-gaggggccatccacagtcttct-3′ (reverse).

### 4.9. Statistical Analysis

The data are presented as mean ± SEM. One- or two-way ANOVA followed by post hoc Bonferroni correction was used to compare results among multiple groups in animal behavioral testing, DRG outer sheath analysis, cell counting, and gene expression. For comparisons between two groups, an unpaired t-test was applied to assess macrophage density. The Mann–Whitney U test was used to analyze calcium imaging data and cytokine concentrations. A *p*-value less than 0.05 was considered statistically significant.

## 5. Conclusions

Our study demonstrates that CLARIX FLO and its key component, HHP, effectively inhibit inflammation and nociception caused by L5 DRG adhesion. The following conclusions can be drawn:

CLARIX FLO exerts both analgesic and anti-inflammatory effects in the newly developed DRG adhesion model.

CLARIX FLO reduced the thickness of the outer sheath of affected DRG, suppressed the inflammatory microenvironment, and decreased the hypersensitivity of isolectin B4-positive neurons.

CD44 acts as a potential downstream mediator of CLARIX FLO in L5 DRG adhesion-induced neuropathic pain.

Treatment with CLARIX FLO adhering to the DRG inhibited the overexpression of TNFα and TRPV1.

## Data Availability

The raw data supporting the conclusions of this article will be made available by the authors on request. During the preparation of this work, the authors used OpenAI’s ChatGPT (GPT-5) SERVICE in order to revise and polish their English to improve readability and clarity. After using this tool/service, the authors reviewed and edited the content as needed and take full responsibility for the content of the published article.

## Supplementary Materials

The following supporting information can be downloaded at: https://www.mdpi.com/article/10.3390/ijms27073096/s1.

## Glossary

ORC = oxidative regenerative cellulose; DRG = dorsal root ganglion; TNFα = tumor necrosis factor-α; IL-6 = interleukin-6; IL-10 = interleukin-10; TRPV1 = Transient Receptor Potential Vanilloid1; PWTs = paw withdrawal thresholds; PERI = Peripherin; mGAPDH = Mouse glyceraldehyde-3-phosphate dehydrogenase; ASIC3 = Acid-sensing ion channel 3; Nav1.7 = Voltage-gated sodium channel 1.7; Nav1.8 = Voltage-gated sodium channel 1.8; IB4 = isolectin B4; L5 = lumbar 5; CCI = chronic constriction injury; Cap = capsaicin.
