# Supplementary material for: CLARIX FLO Inhibits DRG Adhesion-Induced Neuropathic Pain Through the CD44–TRPV1 Signaling Pathway
Source: Int J Mol Sci. 2026 Mar 28;27(7):3096. doi: 10.3390/ijms27073096 (PMC13073902; doi:10.3390/ijms27073096)
Supplement: Supplementary file 1 [file ijms-27-03096-s001.zip › ijms-4168072-supplementary - revised.pdf]

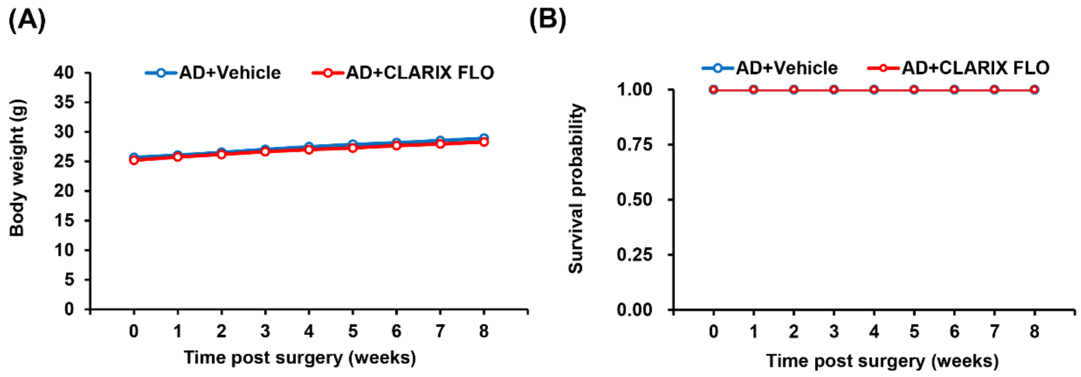

Suppl Figure S1. CLARIX FLO does not affect the normal physiological phenomena of mice. (A) The body weight of mice and (B) the proportion of live mouse individuals were observed for 8 weeks after surgery. n = 6 male mice for each group.

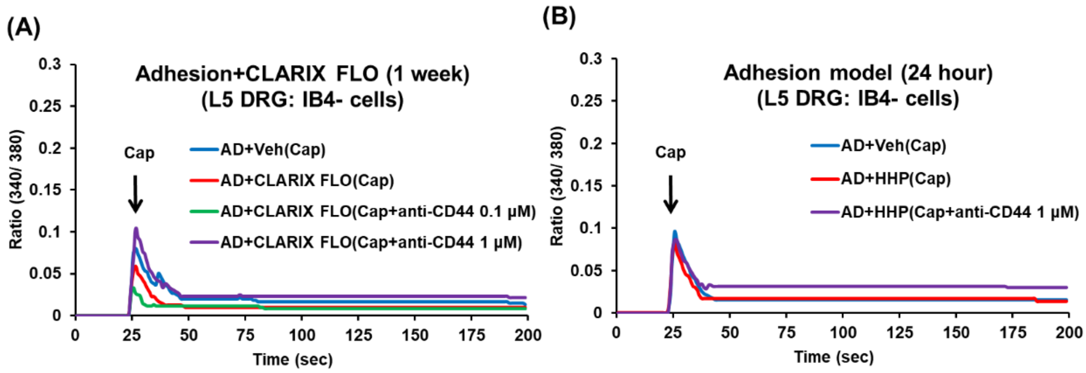

Suppl Figure S2. CLARIX FLO and HC-HA/PTX3 have no effects on IB4-negative L5 DRG neurons. The ratio of 340 nm/ 380 nm represented the cytosolic calcium level of IB4- cells before and after capsaicin (Cap, 0.3 μM) stimuli. (A) One week after treatment with CLARIX FLO, the adhered L5 DRG was taken from mice and the average calcium signaling of IB4-negative cells was observed and calculated; (B) 24 hours after treatment with HHP, the adhered L5 DRG was taken from mice and the average calcium signaling of IB4-negative cells was observed and calculated.
